# Neurophysiological markers of cholinergic dysfunction in pre‐symptomatic autosomal dominant Alzheimer´s disease using Transcranial Magnetic Stimulation (TMS): research design and preliminary results

**DOI:** 10.1002/alz70861_108702

**Published:** 2025-12-23

**Authors:** Daniel A Jiménez, Jose Manuel Matamala

**Affiliations:** ^1^ Hospital del Salvador, Santiago, Santiago Chile; ^2^ Faculty of Medicine, University of Chile, Santiago Chile; ^3^ University of Chile, Santiago, Santiago Chile

## Abstract

**Background:**

Autosomal dominant forms of Alzheimer’s disease (ADAD) represent a small proportion of cases but provide a unique opportunity to track a wide range of pathophysiological changes in the pre‐symptomatic stages of the disease, including brain connectivity dysfunction. Transcranial Magnetic Stimulation (TMS) has proven to be a reliable, non‐invasive technique for assessing intracortical circuits implicated in neurodegenerative disorders. Short‐latency afferent inhibition (SAI), which is primarily mediated by the cholinergic system, has been shown to diminish in the early stages of Alzheimer’s disease (AD).

**Method:**

We developed a TMS protocol to assess cortical excitability and cholinergic function in 14 asymptomatic mutation carriers (PSEN1, PSEN2, or APP genes) and 14 controls. In addition, we plan to perform amyloid PET scans and brain MRI to acquire T1‐weighted 3D sequences for volumetric analysis of the nucleus basalis of Meynert (NbM) using voxel‐based morphometry (VBM) at both the whole‐brain level and predefined regions of interest (with FWE < 0.005).

**Results:**

Here we present our research design and preliminary results in ADAD mutation carriers and controls. We implemented an SAI protocol using various interstimulus intervals (ISIs) relative to the latency of the N20 component of the somatosensory evoked potential of the median nerve (−4, 0, +4, +8 milliseconds). We also recorded measures such as resting motor threshold (RMT), short‐interval intracortical inhibition and facilitation (SICI‐ICF), motor‐evoked potential amplitude and latency (MEP), compound muscle action potential (CMAP), cortical silent period duration (CSP), and central motor conduction time (CMCT).

**Conclusion:**

To our knowledge, this is the first study to use TMS to assess early cortical cholinergic dysfunction in ADAD mutation carriers during the preclinical stages of the disease. We hope that identifying neurophysiological markers of cholinergic dysfunction in the preclinical phases of the AD spectrum will contribute to understanding the mechanisms underlying disease progression from preclinical to clinical stages.

